# Trends in the prevalence, awareness, treatment and control of diabetes in rural areas of northern China from 1992 to 2011

**DOI:** 10.1111/jdi.13095

**Published:** 2019-06-27

**Authors:** Hongyan Zhang, Dongwang Qi, Hongfei Gu, Tao Wang, Yanan Wu, Jingyan Li, Jingxian Ni, Jie Liu, Jun Tu, Xianjia Ning, Jinghua Wang

**Affiliations:** ^1^ Department of Endocrinology and Metabolism Tianjin Medical University General Hospital Tianjin China; ^2^ Department of Neurology Tianjin Haibin People's Hospital Tianjin China; ^3^ Department of Neurology Tianjin Medical University General Hospital Tianjin China; ^4^ Laboratory of Epidemiology Tianjin Neurological Institute Tianjin China; ^5^ Key Laboratory of Post‐Neuroinjury Neuro‐Repair and Regeneration in Central Nervous System Tianjin Neurological Institute Ministry of Education and Tianjin City Tianjin China

**Keywords:** Diabetes mellitus, Epidemiology, Trends

## Abstract

**Aims/Introduction:**

The worldwide prevalence of diabetes mellitus has been increasing over the past decades, particularly in developing countries. Because of the lack of information regarding changes in diabetes mellitus prevalence, awareness, treatment and control in rural China, we assessed these trends – overall and in the context of related health conditions – to explore the impact of these primary health issues on these rates in a poorly educated, rural population.

**Materials and Methods:**

Diabetes mellitus prevalence, awareness, treatment and control rates were compared between two surveys carried out in 1992 and 2011. The residents of three villages, aged 35–64 years, were recruited for this study.

**Results:**

In 1992, 1,091 individuals were interviewed and, in 2011, 2,338 individuals were interviewed. Between the two surveys, the overall diabetes mellitus prevalence in the study population was lower in 1992 than that in 2011 (*P* < 0.001); among men, the prevalence was 5.2‐fold higher in 2011 than in 1992 (10.5 vs 1.7%) and nearly 4.3‐fold higher (11.2 vs 2.1%) among women. Men aged 35–44 years, with >6 years of education, stage I hypertension and being overweight, had a higher prevalence of diabetes mellitus in 2011 than in 1992. Similarly, for the same time periods, there was also a higher diabetes mellitus prevalence among women aged 55–64 years, with 1–6 years of education, stage III hypertension and who were overweight. However, there were no significant changes in diabetes mellitus awareness, treatment or control in this population.

**Conclusions:**

These results suggest that particular efforts must be made to enhance diabetes mellitus prevention, control and public awareness in rural communities in China.

## Abstract 2

The overall prevalence of diabetes mellitus increased significantly both in men and in women over the past two decades. In men, the prevalence of diabetes mellitus increased most significantly for those aged 35–44 years, with >6 years of education, having stage I hypertension and being overweight; but for those aged 55–64 years, with 1–6 years of education, having stage III hypertension and being overweight in women. However, there were no significant changes in diabetes mellitus awareness, treatment or control in this population.

## Introduction

The worldwide prevalence of diabetes mellitus has been increasing over the past few decades, particularly in developing countries^1^. In China, the number of diabetes mellitus patients is expected to increase from 20.8 million (in 2000) to 42.3 million by 2030^2^. In 2013, global statistics estimated that there were 5,096,955 diabetes mellitus‐related deaths among individuals aged 20–79 years, including the 1,271,003 deaths recorded in China^3^. Furthermore, diabetes mellitus and its complications also place a significant financial burden on individuals, families and healthcare systems. In 2015, an estimated $673 billion (USD) was spent on diabetes mellitus‐related health costs, globally, accounting for 12% of total health expenditures^4^. Therefore, early detection and proper management of diabetes mellitus are important steps in reducing the number of deaths and the financial burdens associated with diabetes mellitus. Because China is the world's most populous country, with one‐fifth of the global population, its diabetes mellitus‐associated burden significantly impacts the global situation.

Many studies have described the prevalence, awareness, treatment and control rates of diabetes mellitus^5^, ^6^, ^7^, ^8^. In China, the prevalence of diabetes mellitus is increasing, whereas awareness, treatment and control rates remain quite low^7^, ^8^. As the previous studies have examined different populations and used different criteria, comparing results and assessing changes over time is difficult. Over the past few decades, China has experienced tremendous changes in its socioeconomic and health‐related behaviors, especially in rural areas. Nationally, half of the population lives in rural areas, where income levels and educational attainment are low. Both of these characteristics play decisive roles in increasing the disease burden. Unfortunately, few studies have examined the trends in diabetes mellitus prevalence, awareness, treatment and control in this population^9^. Thus, we assessed the prevalence, awareness, treatment and control trends in rural China, between 1992 and 2011. We examined the overall trends and the impact of related health conditions to explore the interactional effect of these primary health issues on these rates.

## Methods

### Participants

The study population was originally recruited, in 1985, for the Tianjin Brain Study, a population‐based stroke surveillance study in a township in Tianjin, China. The sampling method and the demographic features of the study population were previously reported^10^, ^11^. Briefly, the study included the residents of 18 administrative villages, 95% of whom were farmers with relatively low levels of education and income. The primary source of income was grain production, with an annual per capita income of <$100 in 1991 and <$1,000 in 2010^12^. In 1991, the illiteracy rate for this population, aged 35–74 years, was 30% among men and 40% among women^13^.

### Sampling procedures

We compared diabetes mellitus trends and prevalence using two surveys, one in 1992 and the other in 2011, carried out among adults aged 35–64 years; the methods were also previously described^13^, ^14^. First, the villages were divided into three groups, according to their geographic location (east, south and north); then, we sampled the population from one randomly selected village in each geographic group using a random cluster sampling method. All residents (aged 35–64 years) from the three villages were selected to participate in the survey. In 1992, the total population consisted of 1,456 individuals within the desired age range. From among these, 1,092 residents (75%) participated in the health survey; one individual had a missing blood sample and was excluded from the analysis, leaving 1,091 individuals for inclusion in the analysis.

The 2011 survey originally included 3,007 residents, selected from the same villages included in the 1992 survey. Of these, 2,410 (80%) residents completed both the questionnaire and physical examination (response rate 80.7%); 72 individuals with missing blood samples were excluded, leaving 2,338 individuals to be included in the analyses.

The ethics committee of Tianjin Medical University General Hospital approved the study, and written informed consent was obtained from each resident during recruitment.

### Data collection

All data in the present study were obtained by trained epidemiology researchers who used face‐to‐face interviews to collect information according to a pre‐specified questionnaire. Demographic information, including name, sex, date of birth and education level, were obtained from established records. The participants were categorized into three age groups: 35–44, 45–54 and 55–64 years. Education levels were classified according to the number of years of formal education that the individual had received: illiterate (no formal education), 1–6 years and >6 years. Prior individual and family medical histories, focusing on the presence of hypertension, diabetes mellitus, stroke, transient ischemic attacks and coronary heart disease, were obtained from patient self‐reports or from medical records.

The assessed lifestyle characteristics were cigarette smoking and alcohol consumption habits. Cigarette smoking was defined as smoking more than one cigarette/day for at least 1 year; participants were categorized as non‐smokers, former smokers (ceased smoking for at least 6 months) and current smokers. Alcohol consumption was defined as drinking >500 g of alcohol/week for at least 1 year; participants were categorized as non‐drinkers, former drinkers (temperance for at least 6 months) and current drinkers.

### Measurements

Physical examinations were carried out in the local village clinics during the baseline survey. The examinations facilitated the collection of data regarding blood pressure (BP; including systolic BP and diastolic BP), height and weight. Each participant's body mass index (BMI) was calculated as weight (kg) divided by the square of height (m^2^). Blood samples were also taken to allow determination of fasting plasma glucose (FPG), total cholesterol, triglyceride, high‐density lipoprotein cholesterol and low‐density lipoprotein cholesterol levels; all blood analyses were carried out using enzymatic methods in the central laboratory of the Tianjin Neurological Institute (1992) or at the Ji County People's Hospital (2011).

### Definitions

Hypertension was defined as a systolic BP ≥140 mmHg, diastolic ≥90 mmHg or taking antihypertension medications. Diabetes mellitus was defined as an FPG level ≥7.0 mmol/L, a previous history of diagnosed diabetes, or using insulin or oral antidiabetic drugs^15^. Weight categories were defined as obese (BMI ≥28.0 kg/m^2^) or overweight (BMI 24.0–27.9 kg/m^2^); a BMI of 18.5–23.9 kg/m^2^ was considered normal^16^.

Diabetes mellitus awareness was defined as the participant's self‐reporting of a previous diabetes mellitus diagnosis made by a doctor. Treatment was defined as the use of prescription antidiabetic medications to lower blood glucose levels, at the time of the interview. Diabetes mellitus control was defined as pharmacological treatment of diabetes mellitus resulting in an FPG level <7.0 mmol/L.

### Statistical analysis

Continuous variables are presented as means with standard deviations; between‐group comparisons were carried out using Student's *t*‐test. Categorical variables are presented as numbers and frequencies; χ^2^‐tests were carried out to make between‐group comparisons. The awareness rate was the proportion of individuals with a known history of diabetes mellitus or use of antidiabetic drugs among patients with diagnosed diabetes mellitus. The treatment rate was the proportion of individuals with diagnosed diabetes mellitus who were using antidiabetic drugs. The control rate was the proportion of patients using antidiabetic drugs who had FPG levels <7.0 mmol/L. The rates are presented as percentages with 95% confidence intervals, and the rate differences between the two surveys were compared using χ^2^ testing. *P*‐values <0.05, in two‐tailed tests, were considered statistically significant. SPSS for Windows (version 19.0; SPSS, Chicago, IL, USA) was used for all analyses.

## Results

In 1992, 1,091 individuals (mean age 45.95 years) were interviewed and, in 2011, the mean age of the 2,338 included participants was 54.52 years. In 2011, compared with the 1992 results, the proportions of individuals with >6 years of education (<40%); having hypertension (46.9%) or diabetes mellitus (1.9%); and who were current smokers (25.5%), current drinkers (21.3%), overweight (30.6%) or obese (9.1%) all increased (*P* < 0.001). The mean values of systolic BP (141.07 vs 132.39 mmHg), diastolic BP (86.54 vs 83.83 mmHg), BMI (25.34 vs 23.55 kg/m^2^) and FPG (5.33 vs 4.64 mmol/L) were higher in 2011 than in 1992 (Table 1).

**Table 1:** Description of demographic characteristics among all participants in two surveys

Characteristics	Total		Men		Women	
	1992	2011	1992	2011	1992	2011
Participants (%)	1,091	2,338	423	938	668	1,400
Mean age (SD)	45.95 (9.56)	54.52 (8.20)†	47.36 (9.66)	54.72 (8.41)†	45.05 (9.39)	54.39 (8.06)†
Age group, years (%)		†		†		†
35–44	580 (53.2)	351 (15.0)	200 (47.3)	140 (14.9)	380 (56.9)	211 (15.1)
45–54	238 (21.8)	624 (26.7)	99 (23.4)	235 (25.1)	139 (20.8)	389 (27.8)
55–64	273 (25.0)	1,363 (58.3)	124 (29.3)	563 (60.0)	149 (22.3)	800 (57.1)
Education level (%)	†		†		†	
0 year	280 (25.7)	215 (9.2)	58 (13.7)	19 (2.0)	222 (33.2)	196 (14.1)
1–6 years	444 (40.7)	868 (37.2)	167 (39.5)	300 (32.0)	277 (41.5)	568 (40.7)
>6 years	367 (33.6)	1,250 (53.6)	198 (46.8)	619 (66.0)	169 (25.3)	631 (45.2)
Hypertension (%)	512 (46.9)	1,408 (60.3)†	200 (47.3)	564 (60.3)†	312 (46.7)	844 (60.3)†
Diabetes (%)	21 (1.9)	254 (10.9)†	7 (1.7)	98 (10.5)†	14 (2.1)	156 (11.2)†
Smoking (%)		†		†		†
Never	808 (74.1)	164 (30.1)	165 (39.0)	25 (7.3)	643 (96.3)	139 (69.2)
Ever	5 (0.5)	34 (6.2)	5 (1.2)	34 (9.9)	0	0
Current	278 (25.5)	347 (63.7)	253 (59.8)	285 (82.8)	25 (3.7)	62 (30.8)
Drinking (%)		†		†		†
Never	859 (78.7)	173 (37.6)	204 (48.2)	34 (12.6)	655 (98.1)	139 (72.8)
Ever	0	12 (2.6)	0	12 (4.5)	0	0
Current	232 (21.3)	275 (59.8)	219 (51.8)	223 (82.9)	13 (1.9)	52 (27.2)
BP (mmHg)						
SBP	132.39 (23.66)	141.07 (22.05)†	131.90 (21.43)	141.09 (21.39)†	132.70 (24.98)	141.06 (22.48)†
DBP	83.83 (12.53)	86.54 (13.00)	84.23 (12.18)	87.70 (13.50)	83.59 (12.74)	85.76 (12.61)
BP groups (%)		†		†		†
Normal	594 (54.4)	1,041 (44.5)	231 (54.6)	418 (44.6)	363 (54.3)	623 (44.5)
Stage I	355 (32.5)	807 (34.5)	138 (32.6)	307 (32.7)	217 (32.5)	500 (35.7)
Stage II	74 (6.8)	349 (14.9)	25 (5.9)	147 (15.7)	49 (7.3)	202 (14.4)
Stage III	68 (6.2)	141 (6.0)	29 (6.9)	66 (7.0)	39 (5.8)	75 (5.4)
BMI† (kg/m^2^)	23.55 (3.19)	25.34 (4.60)†	23.23 (2.80)	25.04 (5.48)†	23.74 (3.41)	25.54 (3.88)†
BMI groups (%)		†		†		†
Normal	658 (60.3)	894 (38.2)	281 (66.4)	396 (42.2)	377 (56.4)	498 (35.6)
Overweight	334 (30.6)	952 (40.7)	116 (27.4)	377 (40.2)	218 (32.6)	575 (41.1)
Obesity	99 (9.1)	492 (21.0)	26 (6.1)	165 (17.6)	73 (10.9)	327 (23.4)
Laboratory examinations (mmol/L)						
FPG	4.64 (1.18)	5.33 (1.65)†	4.75 (1.48)	5.30 (1.59)†	4.57 (0.93)	5.34 (1.68)†
TC	4.41 (1.11)	4.78 (1.29)†	4.42 (1.09)	4.70 (1.38)†	4.40 (1.12)	4.83 (1.23)†
TG	1.30 (0.31)	1.65 (0.76)†	1.31 (0.29)	1.75 (0.58)†	1.30 (0.32)	1.58 (1.03)†

^†^Indicated *P* < 0.001 compared between 1992 and 2011. BMI, body mass index; BP, blood pressure; DBP, diastolic blood pressure; FPG, fasting plasma glucose; SBP, systolic blood pressure; SD, standard deviation; TC, total cholesterol; TG, triglyceride.

The overall prevalence of diabetes mellitus in the study population in 1992 was lower than in 2011 (1.9 vs 10.9%; *P* < 0.001); the prevalence among men was 5.2‐fold higher in 2011 than in 1992 (10.5 vs 1.7%) and nearly 4.3‐fold higher (11.2 vs 2.1%) among women. There was also a sex‐related difference reflected in the change in prevalence, by age, between the study periods; there was a 5.4‐fold higher diabetes mellitus prevalence in 2011 than in 1992 (*P* = 0.009) among men aged 35–44 years, but a 4.5‐fold higher prevalence among women aged 55–64 years (*P* < 0.001). However, during that same period, there were no significant changes in diabetes mellitus awareness, treatment or control rates among the study population (Table 2).

**Table 2:** Trends in the sex‐specific prevalence, awareness, treatment and control rates of diabetes by age during 1991 to 2011

	Total			Men			Women		
	1992	2011	*P*	1992	2011	*P*	1992	2011	*P*
Prevalence									
35–44 years	1.2 (0.3–2.1)	4.8 (2.6–7.1)	0.001	1.0 (0–2.4)	6.4 (2.3–10.5)	0.009	1.3 (0.2–2.5)	3.8 (1.2–6.4)	0.075
45–54 years	2.9 (0.8–5.1)	8.7 (6.4–10.9)	0.003	2.0 (0–4.8)	10.6 (6.7–14.6)	0.008	3.6 (0.5–6.7)	7.5 (4.8–10.1)	0.112
55–64 years	2.6 (0.8–5.2)	13.5 (11.7–15.3)	<0.001	2.4 (0–5.2)	11.4 (8.7–14.0)	0.002	2.7 (0.1–5.3)	14.9 (12.4–17.3)	<0.001
Total	1.9 (1.1–2.7)	10.9 (9.6–12.2)	<0.001	1.7 (0.4–2.9)	10.5 (8.5–12.4)	<0.001	2.1 (1.0–3.2)	11.2 (9.6–12.9)	<0.001
Awareness									
35–44 years	57.1 (53.0–61.1)	17.6 (13.7–21.7)	0.134	50.0 (43.0–57.0)	50.0 (41.6–58.4)	0.109	40.0 (35.1–44.9)	12.5 (7.9–16.8)	0.510
45–54 years	14.3 (9.8–18.8)	48.1 (44.1–52.0)	0.121	0	56.0 (49.8–62.6)	0.222	20.0 (13.4–26.9)	41.4 (36.5–46.3)	0.627
55–64 years	57.1 (51.2–63.1)	45.4 (42.8–48.1)	0.705	33.3 (24.7–41.5)	39.1 (35.0–43.1)	1.000	75.0 (68.1–82.2)	48.7 (45.3–52.2)	0.365
Total	42.9 (40.0–45.8)	44.1 (42.1–46.1)	0.913	42.9 (38.1–47.5)	41.8 (38.6–45.0)	1.000	42.9 (39.2–46.7)	45.5 (42.9–48.1)	0.848
Treatment									
35–44 years	25.0 (21.5–28.5)	0	1.000	50.0 (43.0–57.0)	0	1.000	0	0	–
45–54 years	0	26.9 (23.1–30.0)	1.000	–	21.4 (16.0–26.5)	–	0	33.3 (28.7–38.1)	1.000
55–64 years	0	53.0 (50.3, 55.6)	0.055	0	48.0 (43.8, 52.1)	1.000	0	55.2 (51.8, 58.7)	0.102
Total	11.1 (9.2, 13.0)	45.5 (43.5–47.5)	0.076	33.3 (28.8, 37.8)	36.6 (33.5–39.7)	1.000	0	50.7 (48.1–53.3)	0.027
Control									
35–44 years	25.0 (21.5–28.5)	–	–	50.0 (43.0–57.0)	–	–	–	–	–
45–54 years	–	14.3 (11.5–17.0)	–	–	33.3 (27.1– 39.3)	–	–	–	–
55–64 years	–	50.0 (47.3–52.7)	–	–	50.0 (45.9–54.1)	–	–	50.0 (46.5–53.5)	–
Total	11.1 (9.2–13.0)	45.1 (43.1–47.1)	0.072	33.3 (28.8–37.8)	46.7 (43.5–49.9)	1.000	–	44.4 (41.8–47.0)	–

Data is presented as the rate and the 95% confidence interval.

Regardless of education level, the prevalence of diabetes mellitus in 2011 was higher than that in 1992, with it being 4.3‐fold higher among those with 1–6 years of education (*P* < 0.001), overall. Among men, those with ≥7 years of education showed the greatest change in diabetes mellitus prevalence. Among women, the greatest change was observed among those with 1–6 years of education (6.9‐fold higher, *P* < 0.001). However, regardless of education level, there were no significant changes in diabetes mellitus awareness, treatment or control rates in this population (Table 3).

**Table 3:** Trends in the sex‐specific prevalence, awareness, treatment and control rates of diabetes by education levels during 1991 to 2011

	Total			Men			Women		
	1992	2011	*P*	1992	2011	*P*	1992	2011	*P*
Prevalence									
0 year	2.6 (0.7–4.3)	4.8 (1.8–7.5)	0.193	3.3 (0, 8.3)	2.2 (0–5.5)	1.000	2.2 (0.3–4.2)	5.0 (2.0–8.2)	0.118
1–6 years	2.1 (0.7–3.3)	11.2 (9.1–13.3)	<0.001	1.9 (0–3.8)	10.4 (6.9–13.8)	0.001	1.2 (0–2.3)	9.5 (7.1–11.9)	<0.001
>6 years	1.7 (0.3–2.9)	6.7 (5.3–8.0)	<0.001	0.3 (0–1.5)	8.4 (6.2–10.6)	<0.001	3.2 (0.4–5.5)	7.0 (5.0–9.0)	0.053
Awareness									
0 year	1.2 (0–2.3)	2.7 (0.6–5.0)	1.000	0.3 (0–1.6)	2.2 (0–22.6)	1.000	1.3 (0–2.9)	2.6 (0.3–4.8)	1.000
1–6 years	0.6 (0–1.4)	2.9 (1.8–4.0)	1.000	0.6 (0–1.8)	2.9 (1.1–4.9)	1.000	0.3 (0–1.1)	2.9 (1.5–4.2)	1.000
>6 years	0.1 (0–0.2)	2.7 (1.8–3.5)	1.000	0.3 (0–1.5)	3.4 (2.0–4.8)	1.000	0	1.7 (0.7–2.8)	1.000
Treatment									
0 year	0	8.2 (4.6–12.1)	1.000	0	10.9 (0–25.7)	1.000	0	7.5 (3.9–11.4)	1.000
1–6 years	0	16.1 (13.7–18.6)	1.000	0	5.5 (2.8–7.9)	1.000	0	17.7 (14.5–20.7)	1.000
>6 years	25.6 (21.1–30.1)	17.6 (15.4–19.6)	1.000	25.4 (19.1–31.4)	17.9 (14.9–21.0)	1.000	0	14.7 (12.0–17.5)	1.000
Control									
0 year	0	10.9 (6.9–15.4)	1.000	0	0	1.000	0	12.7 (8.0–17.5)	1.000
1–6 years	0	5.7 (4.1–7.2)	1.000	0	5.9 (3.3–8.7)	1.000	0	5.6 (3.7–7.5)	1.000
>6 years	25.6 (21.1–30.1)	12.6 (10.7–14.4)	1.000	25.4 (19.1–31.4)	17.8 (14.8–20.8)	1.000	0	3.2 (1.8–4.5)	1.000

Data is presented as the rate and the 95% confidence interval.

Over the 20‐year study period, the prevalence of diabetes mellitus was significantly higher across the different BP groups, with the exception of those with stage II hypertension. In conjunction with BP increases, the prevalence of diabetes mellitus also increased significantly. The diabetes mellitus prevalence was 1.9‐fold higher for individuals with normal BP (*P* < 0.001), 3.6‐fold higher for individuals with stage I hypertension (*P* < 0.001) and 4.3‐fold higher for individuals with stage III hypertension (*P* = 0.014). However, among men, the greatest change in the prevalence of diabetes mellitus (17.7‐fold higher; 13.1 vs 0.7%, *P* < 0.001) was found among those with stage I hypertension; among women with stage III hypertension, the prevalence was a nearly sixfold higher in 2011 than in 1992 (12.3 vs 1.8%). In addition, regardless of the BP level, there were no significant changes in the diabetes mellitus awareness, treatment or control rates (Table 4).

**Table 4:** Trends in the sex‐specific prevalence, awareness, treatment and control rates of diabetes by blood pressure levels during 1991 to 2011

	Total			Men			Women		
	1992	2011	*P*	1992	2011	*P*	1992	2011	*P*
Prevalence									
Normal	1.9 (0.8–2.9)	5.6 (4.2–7.0)	<0.001	2.3 (0.3–4.1)	6.8 (4.3–9.1)	0.012	1.6 (0.3–3.0)	4.0 (2.5–5.6)	0.041
Stage I	2.2 (0.7–3.8)	10.1 (8.1–12.3)	<0.001	0.7 (0–2.2)	13.1 (9.2–16.9)	<0.001	2.6 (0.6–5.0)	6.9 (4.6–9.0)	0.031
Stage II	4.4 (0–8.7)	10.8 (7.6–14.2)	0.071	3.6 (0–12.3)	7.9 (3.7–12.6)	0.695	4.1 (0–9.8)	9.4 (5.3–13.5)	0.386
Stage III	2.1 (0–4.4)	11.2 (6.0–16.6)	0.014	1.2 (0–2.5)	8.5 (2.0–16.2)	0.173	1.8 (0–7.8)	12.3 (4.5–.19.5)	0.160
Awareness									
Normal	1.1 (0. 3–2.0)	1.6 (0. 9–2.4)	1.000	1.3 (0–2.8)	2.1 (0.8–3.6)	1.000	1.1 (0–2.2)	0.9 (0.1–1.5)	1.000
Stage I	0. 9 (0–1.8)	3.7 (2.4–5.0)	1.000	0	5.6 (3.0–8.1)	1.000	1.1 (0–2.2)	2.9 (1.5–4.5)	1.000
Stage II	0	4.1 (1.9–6.1)	1.000	0	4.0 (0.8–7.3)	1.000	0	3.9 (1.2–6.7)	1.000
Stage III	0	4.1 (0.9–7.6)	1.000	0	3.5 (0–7.3)	1.000	0	4.4 (0–8.5)	1.000
Treatment									
Normal	25.5 (22.1–29.1)	21.1 (18.6–23.6)	0.612	25.4 (19.9–31.2)	22.5 (18.5–26.5)	1.000	0	17.7 (14.8–20.8)	1.000
Stage I	0	33.6 30.3–36.8)	1.000	0	30.7 (25.4–35.8)	1.000	0	15.2 (12.0–18.4)	1.000
Stage II	0	13.9 (10.4–17.7)	1.000	0	5.0 (1.3–8.2)	1.000	–	16.1 (11.2–21.5)	–
Stage III	0	4.9 (1.3–8.6)	1.000	0	–	–	–	7.3 (0.9–12.4)	–
Control									
Normal	25.5 (22.1–29.1)	24.9 (22.3–27.6)	1.000	25.4 (19.9–31.2)	27.7 (23.4–32.1)	1.000	0	5.8 (3.9–7.6)	1.000
Stage I	0	6.4 (4.7–8.1)	1.000	0	2.5 (0.8–4.4)	1.000	0	7.3 (5.1–9.7)	1.000
Stage II	0	7.3 (4.7–10.2)	1.000	0	9.8 (4.7–14.3)	1.000	–	6.5 (3.0–9.8)	–
Stage III	0	6.5 (2.3–10.5)	1.000	0	–	–	–	6.5 (0.9–12.4)	–

Data is presented as the rate and the 95% confidence interval.

The diabetes mellitus prevalence was significantly higher among individuals who were overweight or obese in 2011 than in 1992. Furthermore, the greatest change in diabetes mellitus prevalence was observed for individuals who were classified as overweight (8.4‐fold higher for the total population, including 12‐fold for men and 5.5‐fold for women). As observed for the other analyses, there were no significant changes in diabetes mellitus awareness, treatment or control rates, regardless of BMI (Table 5).

**Table 5:** Trends in the sex‐specific prevalence, awareness, treatment and control rates of diabetes by body mass index during 1991 to 2011

	Total			Men			Women		
	1992	2011	*P*	1992	2011	*P*	1992	2011	*P*
Prevalence									
Normal	2.3 (1.1–3.4)	3.4 (2.3–4.7)	0.208	2.0 (0.4–3.8)	3.6 (1.7–5.4)	0.284	2.1 (0.7–3.6)	3.5 (1.8–5.0)	0.171
Overweight	1.0 (0–1.9)	9.4 (7.5–11.2)	<0.001	0.9 (0–2.6)	11.6 (8.4–14.9)	<0.001	1.0 (0–2.2)	6.5 (4.4–8.4)	0.001
Obesity	4.2 (0.1–8.0)	12.1 (9.3–15.1)	<0.001	2.2 (0–11.8)	12.1 (7.1–17.2)	0.081	5.2 (0.1–10.8)	9.2 (6.0–12.3)	0.764
Awareness									
Normal	1.1 (0.3–1.8)	1.5 (0.8–2.4)	1.000	1.5 (0–2.8)	1.7 (0.5–3.1)	–	1.2 (0–2.1)	1.5 (0.5–2.7)	–
Overweight	0.3 (0–0.9)	3.4 (2.3–4.6)	1.000	0	5.2 (3.0–7.6)	1.000	0.4 (0–1.4)	2.2 (1.0–3.5)	1.000
Obesity	0	4.4 (2.6–6.3)	1.000	0	3.0 (0.4–5.7)	1.000	0	4.0 (1.8–6.1)	1.000
Treatment									
Normal	12.8 (10.2–15.3)	10.6 (8.6–12.7)	1.000	25.4 (20.2–30.4)	15.1 (11.6–18.7)	1.000	0	8.8 (6.3–11.3)	1.000
Overweight	0	20.2 (17.6–22.7)	1.000	0	17.9 (14.1–21.9)	1.000	0	19.8 (16.6–23.1)	1.000
Obesity	0	14.1 (10.9–17.1)	1.000	0	16.0 (10.1–21.4)	1.000	0	13.3 (9.7–17.2)	1.000
Control									
Normal	12.8 (10.2–15.3)	8.1 (6.4–10.0)	1.000	25.4 (20.2–30.4)	6.5 (4.1–9.0)	0.375	0	10.3 (7.6–12.9)	1.000
Overweight	0	5.6 (4.1–7.0)	1.000	0	3.9 (2.0–6.0)	1.000	0	6.1 (4.1–8.0)	1.000
Obesity	0	15.0 (11.9–18.2)	1.000	0	35.6 (28.4–43.1)	1.000	0	4.9 (2.5–7.2)	1.000

Data is presented as the rate and the 95% confidence interval.

## Discussion

To the best of our knowledge, the present study was the first to explore the trends in diabetes mellitus prevalence, awareness, treatment and control in rural areas of northern China. The overall prevalence of diabetes mellitus was 4.7‐fold higher in 2011 than in 1992, 5.2‐fold among men and 4.3‐fold among women. Men, aged 35–44 years, with >6 years of education, stage I hypertension and who were overweight, had a higher prevalence of diabetes mellitus in 2011 than in 1992. There was also a higher diabetes mellitus prevalence in 2011 than 1992 among women aged 55–64 years, with 1–6 years of education, stage III hypertension and who were overweight. Regardless of the increased prevalence of diabetes mellitus, there were no significant changes in diabetes mellitus awareness, treatment or control rates in this population.

According to a World Health Organization report, the age‐standardized global prevalence of diabetes mellitus, in 2014, was approximately twice that in 1980, rising from 4.7 to 8.5% in adults (aged ≥18 years)^17^, including increasing from 4.3% (range 2.4–7.0%) to 9.0% (range 7.2–11.1%) among men, and from 5.0% (range 2.9–7.9%) to 7.9% (range 6.4–9.7%) among women^18^. Another study examined the prevalence of type 2 diabetes mellitus in Samoa between 1978 and 2013. That study showed that the disease prevalence, among men, increased from 1.2 to 19.6% (2.3% increase every 5 years), and from 2.2 to 19.5% among women (2.2% increase every 5 years)^19^. These previous studies suggest that the prevalence of diabetes mellitus has been increasing more rapidly among men than among women in different global populations. Similarly, in the present study, the prevalence of diabetes mellitus was 1.9% in 1992 and 10.9% in 2011 for the overall population, including 1.7% in 1992 and 10.5% in 2011 among men, and 2.1% in 1992 and 11.2% in 2011 among women (all, *P* < 0.001). This apparent sex‐related difference might be partly explained by a higher prevalence of diabetes‐related risk factors (e.g., obesity, hypertension, smoking, alcohol consumption etc.) among men than among women^11^.

Differences in the prevalence of diabetes mellitus among men and women of different ages have also been reported. Men aged <50 years have been reported to have the highest prevalence of diabetes mellitus, whereas women aged >60 years have the highest prevalence of this disease^20^. In the present study, the prevalence of diabetes mellitus also showed the greatest change among men aged 35–44 years (5.4‐fold higher) and among women aged 55–64 years (4.5‐fold higher). These results confirm previous observations that advanced age is a stronger diabetes mellitus risk factor for women than for men^21^.

The observed diabetes mellitus rates have been reported to be significantly higher among individuals with lower education levels than among more educated individuals in a population^22^, for both men and women^23^. In contrast, the present study showed that, regardless of education level, there was a higher prevalence of diabetes mellitus in 2011 than in 1992. Other research has shown that the greatest increase in diabetes mellitus prevalence occurs in people with 1–6 years of formal education. In particular, women with <8 years of formal education had a 1.45‐fold higher risk of developing diabetes mellitus than did more educated women; however, a similar trend was not seen among men^24^. In the present study, in contrast, the greatest changes in diabetes mellitus prevalence were found among men with >6 years of education and among women with 1–6 years of education. The cause of this phenomenon remains unclear and requires further study.

The prevalence of diabetes mellitus has shown an increasing trend, in conjunction with hypertension trends^25^, ^26^, ^27^. In the Women^**’**^s Health Study, hypertensive individuals had twice the risk of developing diabetes mellitus as did those with systolic BPs of 120–129 mmHg^28^. However, in a prospective cohort study of diabetes mellitus risk factors among 7,097 men, an association between baseline BP and diabetes mellitus risk was not observed^29^. In the present study, the prevalence of diabetes mellitus was significantly higher in 2011 than in 1992 across the different BP categories, except for the stage II hypertension group. Elevated BP is also associated with chronic inflammation^30^ and endothelial dysfunction^31^, both of which appear to be mediators of diabetes mellitus risk^32^, ^33^. However, in the present study, the prevalence of diabetes mellitus change was most pronounced in men with stage I hypertension and in women with stage III hypertension. The cause of this phenomenon also remains unclear and requires further study.

Previous data have supported the notion that being overweight (or being obese, in particular) is a strong risk factor for diabetes mellitus in various racial/ethnic populations^34^, ^35^, ^36^. In the current study, the prevalence of diabetes mellitus was observed to increase significantly in specific BMI groups, with the most significant increase being among individuals classified as being overweight. Specifically, diabetes mellitus prevalence was nearly 8.4‐fold higher in 2011 than in 1992 among all overweight participants, including being 12‐fold higher in overweight men and nearly 5.5‐fold higher in overweight women, over the 20‐year study period. Part of the explanation for the increased diabetes mellitus prevalence among overweight individuals is related to the increased insulin resistance and decreased insulin sensitivity associated with elevated BMIs^37^.

Early detection and appropriate management of diabetes mellitus are the most important steps required to reduce diabetic comorbidities and mortality. However, several diabetes mellitus prevalence studies have reported that an increased disease prevalence is associated with awareness, treatment and control rates that are quite low^5^, ^38^, ^39^. The prevalence of diabetes mellitus, in 2011, among the target population in rural China was 5.2‐fold higher than in 1992, whereas disease awareness, treatment and control rates did not change significantly. This is of particular concern, because diabetic complications have been detected in individuals with newly diagnosed diabetes mellitus. Some studies have shown that the rate of diabetes mellitus complications is particularly high among individuals with undiagnosed diabetes^40^, ^41^, ^42^. Hence, many studies have recommended adequate glucose level control to effectively prevent the development of diabetic complications^43^, ^44^. The low levels of disease awareness, treatment and control in the study population might result from inadequate access to healthcare and health education in such poorly educated populations.

The present study had several limitations. First, diabetes mellitus diagnoses were based only on FPG levels ≥7.0 mmol/L, which may have underestimated the diabetes mellitus prevalence. The current diagnostic criteria for diabetes include FPG levels, 2‐h postprandial plasma glucose levels, random blood glucose concentrations, and glycated hemoglobin A1c levels above threshold values 45. However, in China, FPG levels are more commonly used than oral glucose tolerance testing for diabetes mellitus screenings 46. FPG‐based screening for diabetes mellitus, without oral glucose tolerance testing, underestimates the true results, especially in East Asian populations.^47^, ^48^ Furthermore, relying on self‐reported histories of diabetes mellitus, in this poorly educated population, might have also contributed to an underestimation of the number of individuals with diabetes mellitus. Second, the study population was from a single township in China; thus, the findings might not extend to the overall population of China.

The present study reported trends in diabetes mellitus prevalence, awareness, treatment and control over a 20‐year period. There was a higher diabetes mellitus prevalence in 2011 than in 1992, whereas rates of treatment, awareness and control did not show any significant changes. Thus, the study results suggest that diabetes mellitus knowledge among poorly educated people needs to be strengthened, and that BP control and weight management need to be emphasized, especially among young men. Combined, these steps are crucial to reducing the burden of diabetes mellitus in China.

## Disclosure

The authors declare no conflict of interest.
